# Cell Wall Composition and Structure Define the Developmental Fate of Embryogenic Microspores in *Brassica napus*

**DOI:** 10.3389/fpls.2021.737139

**Published:** 2021-10-06

**Authors:** Carolina Camacho-Fernández, Jose M. Seguí-Simarro, Ricardo Mir, Kim Boutilier, Patricia Corral-Martínez

**Affiliations:** ^1^Cell Biology Group, COMAV Institute, Universitat Politècnica de València, Valencia, Spain; ^2^Bioscience, Wageningen University and Research, Wageningen, Netherlands

**Keywords:** androgenesis, arabinogalactan proteins, callose, cell wall, cellulose, microspore embryogenesis, subintinal layer, cell totipotency

## Abstract

Microspore cultures generate a heterogeneous population of embryogenic structures that can be grouped into highly embryogenic structures [exine-enclosed (EE) and loose bicellular structures (LBS)] and barely embryogenic structures [compact callus (CC) and loose callus (LC) structures]. Little is known about the factors behind these different responses. In this study we performed a comparative analysis of the composition and architecture of the cell walls of each structure by confocal and quantitative electron microscopy. Each structure presented specific cell wall characteristics that defined their developmental fate. EE and LBS structures, which are responsible for most of the viable embryos, showed a specific profile with thin walls rich in arabinogalactan proteins (AGPs), highly and low methyl-esterified pectin and callose, and a callose-rich subintinal layer not necessarily thick, but with a remarkably high callose concentration. The different profiles of EE and LBS walls support the development as suspensorless and suspensor-bearing embryos, respectively. Conversely, less viable embryogenic structures (LC) presented the thickest walls and the lowest values for almost all of the studied cell wall components. These cell wall properties would be the less favorable for cell proliferation and embryo progression. High levels of highly methyl-esterified pectin are necessary for wall flexibility and growth of highly embryogenic structures. AGPs seem to play a role in cell wall stiffness, possibly due to their putative role as calcium capacitors, explaining the positive relationship between embryogenic potential and calcium levels.

## Introduction

During the development of the male gametophyte (microsporogenesis and microgametogenesis), cells go through several stages before becoming mature, functional pollen grains. After meiosis, four young microspores are released from the tetrad. Microspores grow and mature and eventually divide asymmetrically to give rise to pollen grains, within which the male gametes will be produced. This natural pathway can be altered when microspores/pollen are cultured *in vitro*. Microspore embryogenesis is an *in vitro* process whereby microspores are induced to become microspore-derived embryos. There are several ways to induce microspore embryogenesis, all of which rely on the application of a stress treatment (Seguí-Simarro et al., 2021). *Brassica napus* is considered a model species to study this process, since most of the knowledge gained in the last decades comes from the use of this species to investigate molecular, cellular, physiological, and developmental aspects of microspore embryogenesis (Jouannic et al., 2001; Corral-Martínez et al., 2013; Soriano et al., 2013; Rivas-Sendra et al., 2017, 2019; Corral-Martinez et al., 2020). In *B. napus* and many other species, microspore embryogenesis is triggered by the application of a heat stress treatment to isolated microspores. This treatment is associated with a series of cellular changes at different levels, involving the cytoskeleton, nucleus, cytoplasm, organelles, and cell wall (Simmonds and Keller, 1999; Seguí-Simarro and Nuez, 2008; Seguí-Simarro et al., 2011; Corral-Martínez et al., 2013; Parra-Vega et al., 2015a; Rivas-Sendra et al., 2019).

The cell wall is not only the protective layer of a plant cell, but also its connection with the environment and neighbor cells, as well as the mechanical force that compensates cell turgor and keeps cells firmly attached. Plant cell walls are formed from different polysaccharides, including pectin, hemicellulose, and cellulose. In different cell types, cell walls may have different proportions of these polysaccharides that define their cell properties and reflect their specific roles. Different polysaccharides can establish different physical and chemical interactions with each other, thereby conferring different properties to the wall. Thus, a combined analysis of the different cell wall components is needed to obtain a good overview of the types and functions of different cell walls.

Pectin is a polymer of galacturonic acid, represented by three major types: homogalacturonan (HG), and rhamnogalacturonan-I (RH-I) and -II (RH-II). They are synthesized in the Golgi system and then packed into vesicles and transported along the actin cytoskeleton to the cell wall, where they form the middle lamella separating adjacent cell walls. Pectin levels and modifications in the middle lamella are crucial for the regulation of cellular adhesion (Bou Daher and Braybrook, 2015). The stiffness of cell walls is also regulated by the pectin composition and level of esterification and by the presence of calcium and cell wall-remodeling enzymes like pectin methylesterases or polygalacturonases (Palin and Geitmann, 2012; Bou Daher and Braybrook, 2015). Cellulose is a linear polymer that is initially required for cell plate stabilization, and then for maintenance of cell wall shape and rigidity (Chen et al., 2018). Arabinogalactan proteins (AGPs) are a large family of heavily glycosylated hydroxyproline-rich cell surface proteins that are present in all higher plants and involved in many different aspects of plant growth and development, including differentiation, cell division, reproduction and, specifically during embryogenesis, cell survival, and embryo patterning (Cheung and Wu, 1999; Gaspar et al., 2001; Seifert and Roberts, 2007; Pereira et al., 2015). Several studies have revealed their involvement in plant reproduction, but their precise role is still unknown. In 2013, a new model about the role of periplasmic AGPs proposed them as calcium capacitors that control the release of calcium to the cytoplasm for cell signaling. Callose is a polymer that is involved in different plant processes, including sexual reproduction, where it is present at different stages. During microsporogenesis, callose is first present in the cell walls of the tetrad, young microspore, generative cell, beneath the intine at the aperture regions of mature pollen grains and at the pollen tube tip and plugs formed during pollen tube growth (Gorska-Brylass, 1967; Dong et al., 2005; Chebli et al., 2012). However, the most general role of callose relates to cell wall formation, where it is essential for cell plate establishment (Seguí-Simarro et al., 2008). All these cell wall components are sequentially added during cell wall biogenesis. The first component transported to the nascent cell plate is HG-pectin. Next, callose is locally synthesized for cell plate expansion. Then, callose is removed in parallel with the onset of cellulose biosynthesis and transport of hemicellulose. Cellulose combines with hemicellulose to create a cellulose–hemicellulose network, while pectin reorganizes into the middle lamella (Albersheim et al., 2011).

During microspore embryogenesis, the cell wall must adapt from covering a single cell, the microspore, to cover a larger, multicellular structure. It seems reasonable to predict that such adaptation requires profound changes in cell wall structure and composition. During the earliest stages of microspore embryogenesis, the sequence of steps described above and the mechanisms of synthesis and/or deposition of cell wall components are altered, resulting in the transient formation of abnormal, irregular, and fragmented cell walls separating the first divided cells, which has profound consequences in further embryo development (Parra-Vega et al., 2015b). These changes are paralleled by altered levels of xyloglucans, pectin, and AGPs (Corral-Martínez et al., 2019), and by the formation of a unique, callose-rich cell wall beneath the intine of the microspore, the subintinal layer (Parra-Vega et al., 2015b). The subintinal layer was proposed to act as a transient physical barrier that protects embryogenic microspores from osmotic changes in the surrounding *in vitro* environment (Rivas-Sendra et al., 2019). Although the specific role of AGPs in microspore embryogenesis is not known, they differentially accumulate in the newly formed cell walls (inner walls and subintinal layer) of induced microspores (Corral-Martínez et al., 2019) and are important for embryo viability and development (Borderies et al., 2004; Corral-Martínez and Seguí-Simarro, 2014).

It is widely accepted that microspore cultures are heterogeneous systems where different cell populations coexist (Seguí-Simarro and Nuez, 2008). Some microspores are not induced and become developmentally arrested or die. Others follow a gametophytic-like development, becoming pollen-like structures that express many of the features of mature pollen grains (Satpute et al., 2005) that develop *in planta*. A third group of microspores develop as embryos. However, not all these embryogenic structures are similar. During zygotic embryogenesis, embryos comprise an embryo proper and a transient basal structure called the suspensor (Dresselhaus and Jürgens, 2021). Previous studies in *B. napus* showed the presence of exine-enclosed (EE), suspensorless structures, and suspensor-bearing structures, both described as embryogenic based on the expression of embryo-reporter genes (Soriano et al., 2013, 2014), and two types of embryogenic callus-like structures, loose and compact (Telmer et al., 1995; Li et al., 2014; Soriano et al., 2014). Recently, a time-lapse imaging study defined the developmental fate of these structures (Corral-Martinez et al., 2020) and distinguished two main groups of microspore-derived structures according to their ability to form differentiated embryos. EE structures and suspensor-bearing embryos (SUS) are considered highly embryogenic because a high proportion of them develop into differentiated embryos, while only a low percentage become callus-like structures or die. Compact callus (CC) and loose callus (LC) structures are considered barely embryogenic because they have a very low viability and ability to form differentiated embryos.

Due to this remarkable heterogeneity and the well-known role of cell wall in shaping *in vitro* morphogenesis, a new approach for understanding cell wall dynamics in each type of microspore-derived structure is required to determine the relationship between their cell wall characteristics and their competence for the successful completion of embryo development. In this work, we performed a detailed study of the cell wall structure and composition in the different structures found in *B. napus* microspore cultures, focusing on pectin (both highly and low methyl-esterified), callose, and AGPs, using confocal and transmission electron microscopy (TEM) and immunogold labeling. Our results uncover a relationship between cell wall composition and structure and the developmental fate of each type of microspore-derived structure, providing new insights on the role of these cell wall components during microspore embryogenesis. This study also provides a broader framework for understanding cell fate and morphogenic processes during *in vitro* embryogenesis.

## Materials and Methods

### Plant Material

DH4079 plants were used as donor plants for microspore culture. DH4079 is a doubled haploid line derived from *B. napus* L. cv. Topas, and highly responsive to induction of microspore embryogenesis. Plants were grown continuously at 18°C in growth chambers in 20 cm pots at 60% relative humidity under a 16/8 h photoperiod until just before flowering, and then transferred to 10°C/5°C (day/night) with the same photoperiod.

### Microspore Culture

Flower buds containing a majority of late, vacuolated microspores and early bicellular pollen grains were collected from donor plants. The isolation, induction, and culture was performed according to Corral-Martínez et al. (2021). Flower buds were surface sterilized with 4 g/L sodium hypochlorite and then washed three times with sterile water. Microspores were isolated from anthers by crushing buds with a sterile syringe piston in isolation medium (13% w/v sucrose in dH_2_O, pH 5.8) and filtering the suspension through a 30 μm nylon mesh (Millipore). This was followed by three rounds of centrifugation (100 × *g*, 4 min each) and resuspension of the microspores in isolation medium. After the last centrifugation, microspores were resuspended in culture medium (NLN-13 medium), made with NLN basal medium (Duchefa, Netherlands), 13% (w/v) sucrose, and 0.5 g/L Ca(NO_3_)_2_⋅4H_2_O, pH 5.8. Microspore density was adjusted with a hemocytometer (Camacho-Fernández et al., 2018) to 40,000 microspores/ml with culture medium. Microspores were plated in 6 cm tissue culture dishes (3 ml suspension per plate) and incubated in darkness for 1 day at 32°C. Afterward, plates were kept at 25°C in darkness for progression of embryogenesis.

### Staining and Observation by Confocal Laser Scanning Microscopy

*Brassica napus* microspore cultures were collected at days 0, 2, 5, and 8 after culture initiation and fixed overnight at 4°C with 4% paraformaldehyde in PBS (pH 7.4). Afterward, samples were washed three times with PBS and then stored at 4°C in 0.1% paraformaldehyde in PBS until use. Staining of callose and cellulose in microspores was performed according to Parra-Vega et al. (2015b) and Rivas-Sendra et al. (2019). For callose detection, samples were incubated first with 10 μg/ml propidium iodide (PI; Fluka) for 10 min, and then with 0.1% aniline blue (AB; Fluka) for 20 min. PBS was used for preparation of all staining solutions and for the three washes after each staining. Samples were mounted in 17% Mowiol 4-88 (Sigma-Aldrich) and 33% glycerol (v/v) in PBS. For cellulose detection, samples were stained with 0.01% Direct Red (Sigma) in 0.1 M PBS for 30 min, washed three times with PBS, mounted in a 1:1 mix of Mowiol and 2.5 μg/ml 4′,6-diamidine-2′-phenylindole dihydrochloride (DAPI; Sigma-Aldrich) prepared as described in Custers (2003), and then incubated for at least 15 min. Fluorochromes were excited with 405 nm (for DAPI and aniline blue) and 561 nm (for PI and Direct Red) laser lines, and emission was recorded between 450–490 and 580–650 nm, respectively. All the samples were incubated in darkness and observed with a ZEISS 780 Axio Observer confocal laser scanning microscope. Images were processed with Zeiss Efficient Navigation (ZEN) and FIJI software (Schindelin et al., 2012).

To observe cell wall thickness, the fixed samples were stained with Direct Red as described above and with SCRI Renaissance Cell 2200 (SR2200). Staining of SCRI Renaissance 2200 was performed according to Musielak et al. (2015), incubating samples 30 min before observation. SR2200 was excited with a 405 nm laser line and emission was recorded between 415 and 476 nm. At least 100 embryogenic structures were examined with a Leica SP5 confocal laser scanning microscope. Images were processed with Leica Application Suite Advanced Fluorescence (LAS AF) and FIJI software (Schindelin et al., 2012).

### Processing of Samples for Transmission Electron Microscopy

Five-day-old cultured microspores were collected for TEM. Processing was performed as described in Seguí-Simarro and Nuez (2007) with some modifications. Samples were fixed in Karnovsky fixative (Karnovsky, 1965), post-fixed with 2% OsO_4_ in 0.05 M cacodylate buffer and washed three times with 0.025 M cacodylate. Microspores were immobilized and concentrated as follows: cacodylate buffer was removed and 1–2 drops of warm (liquid) 15% gelatin in cacodylate buffer were added. Microspores were then resuspended in the liquid gelatin, centrifuged (1 min at 125 × *g*) and allowed to cool on ice for gelatin solidification. Once solid, 20 μl of 1% paraformaldehyde in cacodylate buffer was added, and the samples stored overnight at 4°C. Finally, gelatin-embedded samples were cut in small pieces and kept in cacodylate buffer until use. Samples were dehydrated in a progressive methanol series and embedded and polymerized in Embed 812 resin (Electron Microscopy Sciences). Ultrathin (80 nm) sections were obtained from at least three different blocks of each sample with a Leica UC6 ultramicrotome for TEM observation. Sections were mounted on carbon and formvar-coated, 200-mesh nickel grids (Electron Microscopy Sciences), stained with uranyl acetate in 70% methanol (6 min) and lead citrate (30 s). Images were taken with a Jeol JEM 1010 TEM. For morphometric studies of the cell wall, the minimum number of images required was determined using the progressive mean test (Williams, 1977) according to Corral-Martínez et al. (2019). For each type of structure, the width of the intine and subintinal layer of the cell walls was measured using FIJI software (Schindelin et al., 2012). Statistically significant differences in cell wall and subintinal layer width were determined using a least significant difference (LSD) test with *p* ≤ 0.05.

### Immunogold Labeling

For callose detection, an anti-callose monoclonal antibody (mAb) from Biosupplies (Australia) was used that specifically recognizes linear (1→3)-β-oligosaccharide segments in (1→3)-β-glucan. For AGP detection, a JIM13 rat IgM mAb (PlantProbes, United Kingdom) was used that recognizes the AGP2 epitope present in different plant exudates like gum arabic and gum ghatti (Knox et al., 1991). For pectin detection, the following antibodies (PlantProbes) were used: JIM7, a rat IgA mAb that cross-reacts with highly methyl-esterified epitopes of the homogalacturonan domain of pectic polysaccharides (Knox et al., 1990; Willats et al., 2000) and JIM5, a rat IgG mAb that cross-reacts with low methyl-esterified epitopes of homogalacturonan (Knox et al., 1990; Willats et al., 2000). For immunolocalization of cell wall components, sections were hydrated with PBS for 1 min. Non-specific binding was prevented by incubation with 0.2% BSA and 3% skimmed milk in PBS for 30 min. For the anti-callose antibody, sections were incubated with the primary antibody diluted 1:5000 in 1% BSA in PBS for 1 h. For the remaining antibodies, sections were incubated with the primary antibody diluted 1:5 (JIM13) or 1:2 (JIM7 and JIM5) in 0.2% BSA in PBS for 1 h. For all antibodies, sections were then washed six times during 5 min with 0.2% BSA in PBS. Secondary antibodies (goat anti-mouse for anti-callose antibody and goat anti-rat for the rest) conjugated with 10 nm gold particles (BBI solutions, United Kingdom) were diluted 1:25 with 0.2% BSA in PBS (1% BSA for the anti-callose antibody) and incubated with the sections for 45 min. Finally, sections were washed five times with PBS, 5 min each, post-fixed with 2% glutaraldehyde in PBS for 10 min and washed again with PBS and distilled water as the last step. Samples were then counterstained with uranyl acetate in 70% methanol (6 min) and lead citrate (30 s). Controls excluding primary antibodies were performed to check for non-specific binding of the secondary antibody. All steps were performed at 25°C.

### Quantification of Immunogold Labeling

Three different blocks were randomly selected and micrographs were taken systematically at the same magnification. Several micrographs were taken randomly from all the structures of interest on each grid. The minimum number of micrographs was determined using the progressive mean test (Williams, 1977), with a minimum confidence limit of α = 0.05. In general, around 15–20 micrographs per antibody and type of structure were studied. The labeling density of each compartment under study was manually calculated by counting the number of particles and dividing this by the area where they were counted, being expressed as particles/μm^2^. For all the structures studied in this work, the analyzed compartments were (1) Golgi stacks and cytoplasmic vesicles, (2) inner cell walls, and (3) outer cell walls (the intine and the subintinal layer formed in microspore-derived structures) but excluding the outermost exine layer. Particles over regions where the presence of the studied epitopes is not expected (the cytoplasm excluding Golgi stacks and vesicles, cytoplasmic organelles, nuclei, and the embedding resin outside cells), were considered as background noise, which was estimated as the particle density of these regions. The area in μm^2^ was measured using a square lattice composed of 11 × 16 squares of 15 × 15 mm each. Labeling density was expressed as the average labeling density of all micrographs ± SD. Comparisons of mean labeling densities were performed by one-way ANOVA tests using StatGraphics software. In cases where data were not homoscedastic, Mood’s median test was used. Means were separated using a LSD test with *p* ≤ 0.05. Histograms of the same antibody in different subcellular compartments were built using the same *Y*-axis scale to facilitate comparisons.

## Results

### DH4079 Microspore Cultures Comprise a Heterogeneous Population of Structures That Respond Differently to Embryogenesis Induction

**FIGURE 1:**
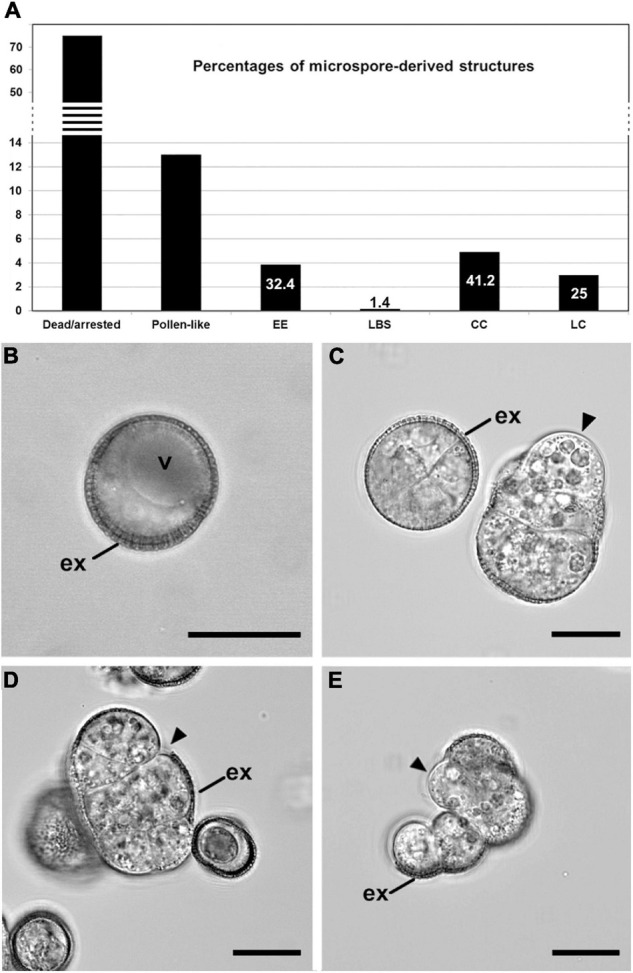
Development of isolated microspore cultures. **(A)** Percentages of the different structures derived from *in vitro* cultured microspores, including dead or arrested microspores, pollen-like structures, exine-enclosed (EE) structures, loose bicellular structures (LBS), compact calli (CC), and loose calli (LC). Percentages were calculated from a total of 1245 randomly selected structures. The numbers in EE, LBS, CC, and LC represent the relative percentages of each type of potentially embryogenic structure, excluding non-embryogenic forms (dead/arrested and pollen-like). **(B–E)** Confocal microscopy images of a vacuolated microspore at the start of culture (day 0; **B**), an EE structure (**C** left), a LBS (**C** right), a CC **(D)**, and a LC **(E)**. Arrowheads point to areas devoid of exine (ex). Bars: 20 μm.

During *B. napus* microspore culture, not all microspores are equally sensitive to heat stress and therefore follow different pathways. The majority of microspores either followed a gametophytic-like pathway and became pollen-like structures or arrested in development and eventually died. Depending on the genotype and culture conditions, microspore embryogenesis may occur in a variable percentage of microspores. Our study focused on the highly responsive DH4079 line, where the only embryogenic structures previously described were EE and suspensor-bearing (SUS) embryos (Telmer et al., 1995; Supena et al., 2008). These cell types together with CC and LC structures were described in the low responsive DH12075 genotype. As it was not clear whether the presence of CC and LC in microspore cultures is specifically associated with the recalcitrance of this genotype, we reexamined the types of structures found in DH4079 cultures in quantitative (Figure 1A) and qualitative detail (Figures 1B–E). The starting point of this analysis, before embryo induction, was a population comprising mostly vacuolated microspores (Figure 1B) and some young pollen grains. After the heat stress treatment, 75% of the initial microspores/pollen died or arrested and 13% followed a pollen-like development. A third subset (12%) comprised four types of multicellular, proliferating and potentially embryogenic structures with different sizes, levels of cellular organization and adhesion, and degrees of exine coverage. EE structures (Figure 1C left; 3.85%) were highly compact and organized (defined as having straight cell walls and regular division patterns), fully covered by exine, with thin inner cell walls and closely adhered cells. CC structures (Figure 1D; 4.9%) were similar to EE but more disorganized, less compact, with thicker inner cell walls, less cellular adhesion and more extensive exine breakage. LC (Figure 1E; 2.97%) were very disorganized structures with weak cellular adhesion where the exine was either completely ruptured or absent. Finally, loose bicellular structures (LBS) (Figure 1C right; 0.16%) comprised 2–3 loosely connected cells surrounded by partially ruptured exine, but with a level of cell organization higher than LC and similar to EE. These structures strongly resembled the few-celled suspensors observed in DH4079 under milder heat stress treatment (Supena et al., 2008) or in DH12075 (Corral-Martinez et al., 2020). However, in the absence of cell fate tracking data, we refer to these as LBS.

Our observations indicate that the same embryogenic cell types found in the low embryogenic DH12075 line are also found in the highly response DH4079 line and therefore, that the heterogeneity of embryogenic cell types in DH4079 has been previously underestimated. Given the extensive cell wall remodeling that takes place as microspores and pollen transition to embryos, we examined whether these four types of embryogenic structures also showed differences in their cell wall architectures.

### External Cell Walls Have a Different Width in Different Types of Proliferating Structures

**FIGURE 2:**
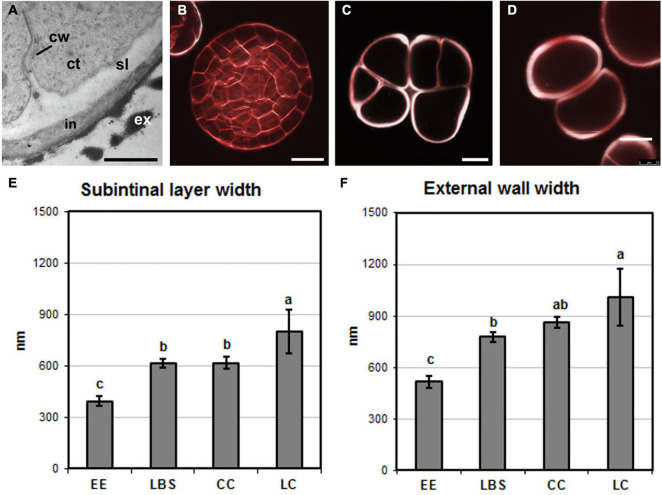
Cell wall width. **(A)** Electron microscopy image of an exine-enclosed (EE) structure, showing the inner cell wall (cw), subintinal layer (sl), intine (in), and exine (ex). ct, cytoplasm. **(B–D)** Images of structures stained with SCRI Renaissance Cell 2200 observed by confocal microscopy. **(B)** Young, EE-derived embryo, **(C)** compact callus (CC), and **(D)** loose callus (LC). **(E)** Subintinal layer width (in nm) in each type of structure. **(F)** Width (in nm) of the intine + subintinal layer in each type of structure. Different letters represent significant differences according to the LSD test. Bars: **(A)**, 500 nm; **(B–D)**, 10 μm.

In DH12075, it was previously shown that callus-like structures (LC and CC) have thicker cell walls than EE (Corral-Martinez et al., 2020). We therefore used SCRI Renaissance Cell 2200 staining to examine cell wall thickness in the DH4079 proliferating structures, paying special attention to the two cell wall layers observed previously in embryogenic microspores, the subintinal layer and the intine (Figure 2A). Highly embryogenic EE structures (Figure 2A) and the young globular embryos derived from them (Figure 2B) had cell walls that were thinner than those of barely embryogenic structures (CC and LC, Figures 2C,D, respectively). To quantify cell wall thickness, we measured both the subintinal layer and the total cell wall width in TEM images. We observed that the cell wall of callus-like structures (CC and LC) was wider than that of highly embryogenic structures (EE and LBS; Figure 2E). Consistent with this, the width of the subintinal layer was also higher in barely embryogenic structures (Figure 2F), and proportional to the total cell wall width, which was always around 70–80%, with no significant differences among structures. In summary, there were significant differences between the cell wall widths of the different DH4079 microspore-derived structures, with barely embryogenic structures having thicker walls than highly embryogenic structures. By contrast, the percentage of subintinal layer relative to the entire cell wall was similar for the different structures, which suggested that the differences in cell wall dynamics between structures are not related to differences in the relative thickness of the subintinal layer.

### Exine-Enclosed and Loose Bicellular Structures Accumulate More Callose in Internal and External Cell Walls

**FIGURE 3:**
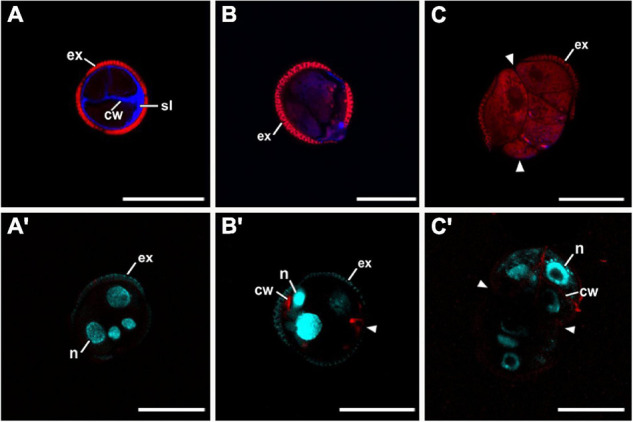
Confocal microscopy images of EE structures at 2 **(A,A′)**, 5 **(B,B′)**, and 8 days of culture **(C,C′)**, stained with aniline blue for callose (blue in **A–C**) together with propidium iodide (red), and with Direct Red for cellulose (red in **A′–C′**) together with DAPI (blue). Arrowheads indicate regions of exine (ex) rupture or absence. cw, cell wall; n, nucleus; sl, subintinal layer. Bars: 20 μm.

**FIGURE 4:**
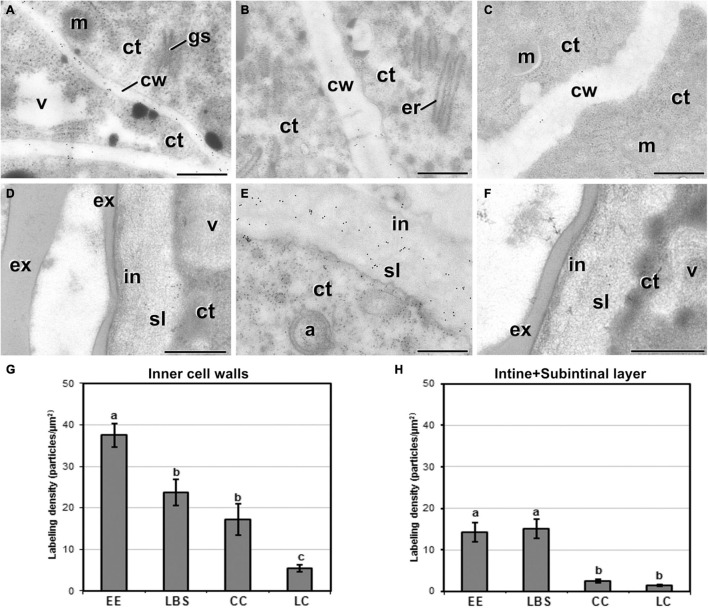
Callose detection by immunogold labeling and quantification in TEM images of 5-day-old microspore cultures. Inner cell wall images from EE **(A)**, LBS **(B)**, and LC **(C)** structures. Outer cell wall images from EE **(D)**, LBS **(E)**, and CC **(F)** structures. **(G)** Callose labeling density in inner cell walls. **(H)** Callose labeling density in external cell walls. Different letters represent significant differences according to the LSD test. Labeling density is expressed in number of particles per μm^2^. a, autophagosome; ct, cytoplasm; cw, cell wall; er, endoplasmic reticulum; ex, exine; gs, Golgi stack; in, intine; m, mitochondrion; v, vacuole. Bars: 500 nm.

Next, we examined the composition of the cell wall in the four different types of structures. In general, callose staining was present in large amounts in the walls of all structures during the first stages of embryogenesis, but was reduced upon culture progression (Figures 3A–C). Cellulose staining showed the opposite pattern, with faint wall staining during the first embryogenic stages, followed by increased staining at later stages, in parallel with a reduction in callose staining (Figures 3A′–C′). In the anti-callose immunogold labeling experiments, the unspecific labeling density observed in the cytoplasm and organelles (background noise) was consistently low compared with that of cell walls, as expected. No significant differences among structures were observed in cell walls between adjacent cells of the same structure. However, different structure types showed different levels of callose in their cell walls. In highly embryogenic structures (EE and LBS), anti-callose labeling was present in higher amounts than in barely embryogenic structures (LC and CC), in both internal (Figures 4A–C) and external walls (Figures 4D–F), although at different levels. In general, inner cell walls showed more anti-callose labeling than external walls in all types of structures (Figures 4G,H). Statistically significant differences among structure types were found in internal (Figure 4G) and external cell walls (Figure 4H). EE showed a significantly higher callose content in inner cell walls than the rest of the structures, while in LC it was significantly lower than the rest. In external walls (intine + subintinal layer), two groups were observed according to callose labeling density: highly embryogenic structures (EE and LBS) presented higher labeling than barely embryogenic structures (CC and LC), where labeling density was close to zero. Upon embryogenesis induction, callose is known to accumulate only in the subintinal layer (Parra-Vega et al., 2015b), implying that the labeling we observed in outer cell walls can only be attributed to the subintinal layer. Together, our results showed remarkable differences in callose accumulation between the different embryogenic structures: abundant in highly embryogenic structures (EE and LBS) and very limited in barely embryogenic structures (CC and LC). This suggests that differences in callose accumulation in the subintinal layer are related to the level of cellular organization and embryogenic competence of the different structures.

### The Cell Walls of Exine-Enclosed and Loose Bicellular Structures Accumulate More JIM13-Cross-Reacting Arabinogalactan Proteins Than Those of Compact Callus and Loose Callus Structures

**FIGURE 5:**
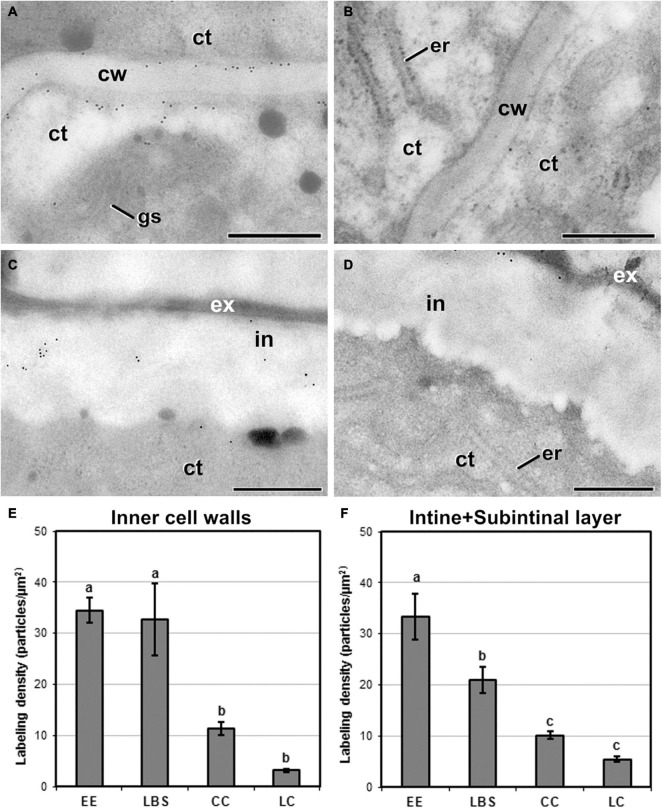
Detection of JIM13 cross-reacting AGPs by immunogold labeling and quantification in TEM images of 5-day-old microspore cultures. Inner cell wall images of EE **(A)** and LC structures **(B)**. Outer cell wall (intine + subintinal layer) images of EE **(C)** and CC structures **(D)**. **(E)** Quantification of JIM13-cross-reacting AGPs in inner cell walls. **(F)** Quantification of JIM13-cross-reacting AGPs in outer cell walls (intine + subintinal layer). Labeling density is expressed as number of particles/μm^2^. Different letters represent significant differences according to the LSD test. ct, cytoplasm; cw, cell wall; er, endoplasmic reticulum; ex, exine; gs, Golgi stack; in, intine; m, mitochondria; v, vacuole. Bars: 500 nm.

We studied the differences in abundance and distribution of JIM13-cross-reacting AGPs in the different structures (Figure 5). JIM13 epitopes were detected in Golgi stacks and cytoplasmic vesicles, although at much lower densities than in cell walls, and with no differences between structures (data not shown). Background noise was always negligible. In inner walls, EE (Figure 5A) and LBS showed higher JIM13 labeling density than CC and LC (Figure 5B). In outer walls, EE (Figure 5C) exhibited a significantly higher JIM13 labeling density than LBS, LC, and CC structures (Figure 5D). The distribution of JIM13 labeling in cell walls was similar to that of callose but, opposite to callose, the JIM13 levels in inner and outer walls of a given structure were similar (Figures 5E,F). Statistically significant differences among structures were found in inner (Figure 5E) and outer walls (Figure 5F), with barely embryogenic structures (LC and CC) having a significantly lower labeling density than highly embryogenic structures (EE and LBS). In summary, AGPs carrying the JIM13 epitope accumulated in a different manner in the cell walls of the different embryogenic structures, being more abundant in highly embryogenic and hardly detectable in barely embryogenic structures.

### Structures With Less Intercellular Adhesion Have Reduced Levels of Low Methyl-Esterified Pectin

**FIGURE 6:**
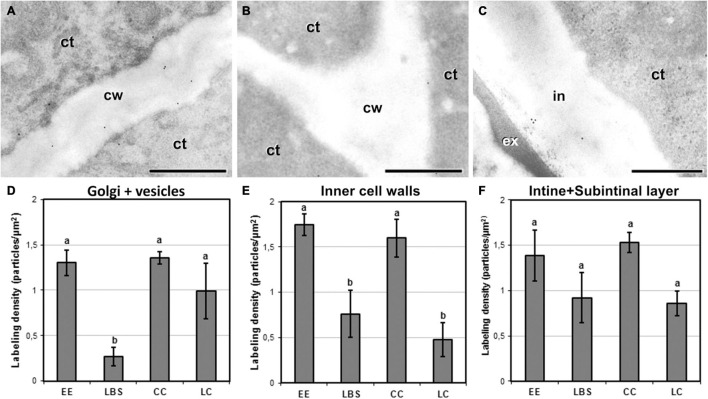
Detection of JIM5 cross-reacting low methyl-esterified pectin by immunogold labeling and quantification in TEM images of 5-day-old microspore cultures. Inner cell wall images of EE **(A)** and LC **(B)**. **(C)** Outer cell wall image of EE. **(D)** Quantification of JIM5 cross-reacting low methyl-esterified pectin in cytoplasmic Golgi stacks and vesicles. **(E)** Quantification of JIM5 cross-reacting low methyl-esterified pectin in inner cell walls. **(F)** Quantification of JIM5 cross-reacting low methyl-esterified pectin in outer cell walls (intine + subintinal layer). Labeling density is expressed in number of particles per μm^2^. Different letters represent statistically significant differences according to the LSD test. ct, cytoplasm; cw, cell wall; ex, exine; in, intine; m, mitochondrion. Bars: 500 nm.

We performed immunogold labeling with JIM5 antibody to determine whether the four different embryogenic structures show differences among structures in the levels of low methyl-esterified pectin (Figure 6). For all the structures, JIM5 signal was present in inner walls and in both the intine and subintinal layer of outer walls, as well as in cytoplasmic Golgi stacks and transport vesicles. Background labeling density was always negligible, irrespective of the sample studied. In general, compact structures such as EE (Figure 6A) and CC presented more JIM5 labeling in inner cell walls than the loose LBS and LC (Figure 6B). This trend was also observed in outer walls (Figure 6C). Quantification of immunogold labeling confirmed these observations. Significant and structure-specific differences in labeling density were observed in the Golgi stacks and cytoplasmic vesicles (Figure 6D), and in the inner walls (Figure 6E). In outer walls (Figure 6F), the pattern observed was remarkably similar to that of inner walls, although the differences observed were not statistically significant. Considering all these results together, LBS showed the lowest density of low methyl-esterified pectin of all types of structures, followed by LC. These results pointed to a relationship between the level of low methyl-esterified pectin and the degree of adhesion between cells of the same structure. In particular, compact structures with higher cell adhesion (EE and CC) showed higher levels of low methyl-esterified pectin than loosely connected structures (LBS and LC).

### Exine-Enclosed and Loose Bicellular Structures Accumulate More Highly Methyl-Esterified Pectin in Their Internal and External Cell Walls

**FIGURE 7:**
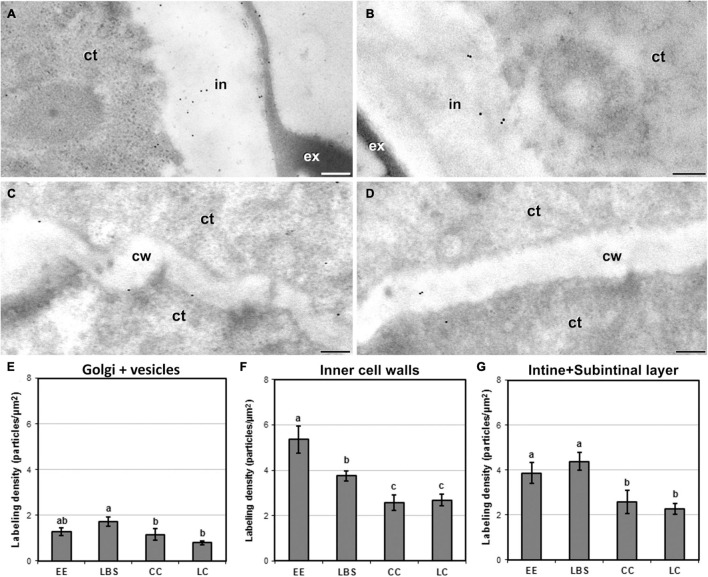
Detection of JIM7-cross-reacting highly methyl-esterified pectin by immunogold labeling and quantification in TEM images of 5-day-old microspore cultures. External cell wall images of EE **(A)** and CC **(B)**. Inner cell wall images of EE **(C)** and CC **(D)**. **(E)** Quantification of JIM7-cross-reacting highly methyl-esterified pectin in cytoplasmic Golgi stacks and vesicles. **(F)** Quantification of JIM7-cross-reacting highly methyl-esterified pectin in inner cell walls. **(G)** Quantification of JIM7-cross-reacting highly methyl-esterified pectin in outer cell walls (intine + subintinal layer). Labeling density is expressed in number of particles per μm^2^. Different letters represent statistically significant differences according to LSD test. ct, cytoplasm; cw, cell wall; ex, exine; in, intine; m, mitochondria. Bars: 200 nm.

JIM7 immunogold labeling revealed that highly methyl-esterified pectin was present in cytoplasmic Golgi stacks and transport vesicles, inner walls and the intine and subintinal layers. Background noise was negligible. Quantitatively, average labeling density in outer and inner walls was similar for each type of structure. Cell walls showed a pattern of JIM7 immunolocalization similar to that of JIM13 and anti-callose: highly embryogenic EE (Figures 7A,C) and LBS structures showed higher labeling densities than barely embryogenic CC (Figures 7B,D) and LC structures. Differences were found between the four structures for both inner and outer walls, as well as for Golgi stacks and vesicles (Figure 7E), where labeling density was lower than in cell walls, showing a pattern similar to outer walls. In inner walls (Figure 7F), JIM7 epitopes were significantly more abundant in highly embryogenic (EE and LBS) structures than in barely embryogenic structures (CC and LC). No differences in JIM7 labeling density were found in outer walls between EE and LBS (Figure 7G), but labeling density in these structures was higher than in LC and CC.

**FIGURE 8:**
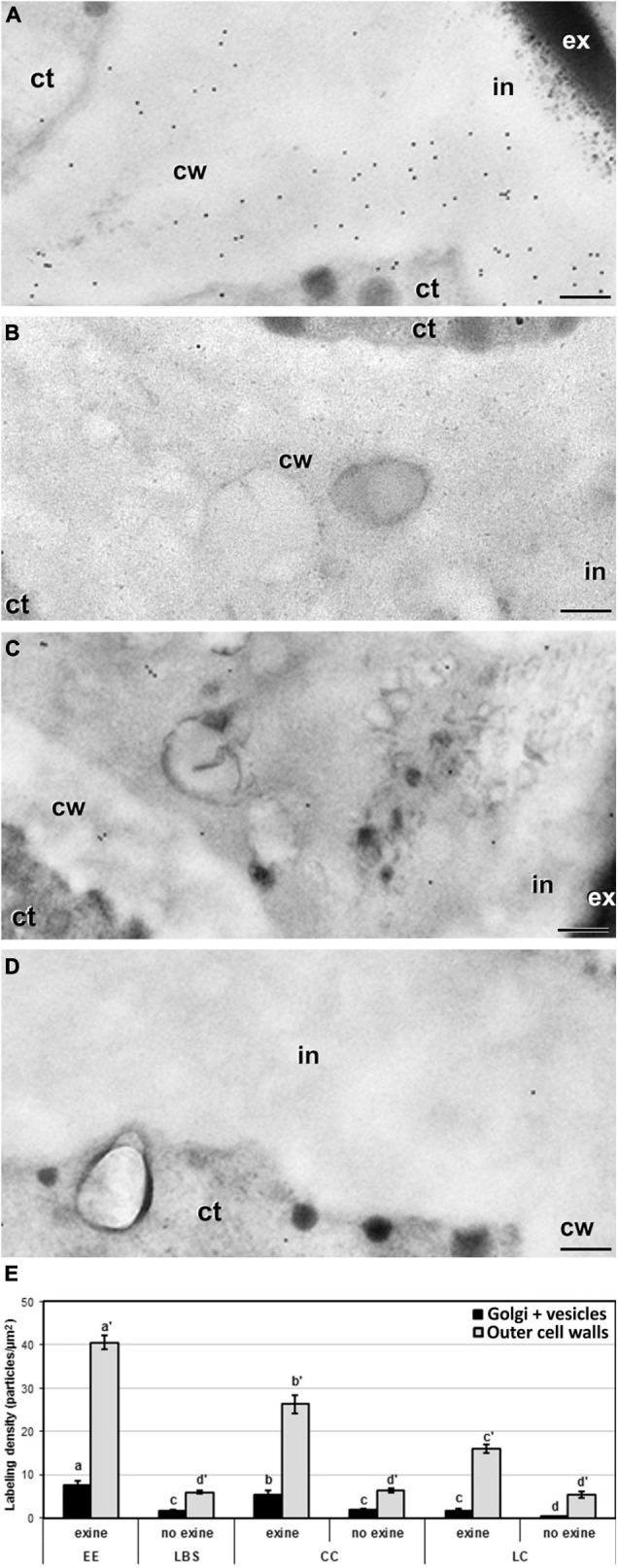
Detection of JIM7 cross-reacting highly methyl-esterified by immunogold labeling and quantification in TEM images of cell wall regions connecting to outer walls from 5-day-old microspore cultures. Images of cell wall regions connecting with outer walls in EE **(A)**, LBS without exine **(B)**, and LC with **(C)** and without exine **(D)**. **(E)** Quantification of JIM7-cross-reacting highly methyl-esterified pectin in cytoplasmic Golgi stacks and vesicles and outer cell wall in different types of structures and in areas with or without exine. Labeling density is expressed in number of particles per μm^2^. Different letters represent statistically significant differences obtained with LSD test. ct, cytoplasm; cw, cell wall; ex, exine; in, intine. Bars: 200 nm.

In addition to the general patterns observed in the quantitative analysis and to the differences observed among different wall and layer types, we detected remarkable differences among structures in the cell walls separating adjacent cells, and in particular, in the outermost region where these walls connect with the subintinal layer and the intine. Depending on the presence or absence of exine covering these regions, the labeling density were dramatic. EE structures, which are completely covered by exine (Figure 8A), showed levels of JIM7 epitopes in these regions that were much higher than in equivalent regions of other structures devoid of exine (Figure 8B). In structures partially covered by exine (CC and LC), the regions covered by exine (Figure 8C) had a much higher labeling than the equivalent regions devoid of exine in the same structure (Figure 8D). Statistical analysis of labeling density (Figure 8E) confirmed these observations. These results suggest that the presence of exine in the regions where inner wall edges connect with outer walls, influences the accumulation of highly methyl-esterified pectin in these regions, being reduced in structures with lower levels of cell compaction and adhesion. Therefore, exine presence appears to play a role in the accumulation of highly methyl-esterified pectin and in the adhesion properties of cell wall edges.

## Discussion

*Brassica napus* microspore cultures are heterogeneous systems where different populations with different developmental fates coexist. It was initially assumed that the subset of embryogenic microspores is homogeneous (Telmer et al., 1995) and follows the same pathway toward embryogenesis. This idea changed when two different types of structures, EE, suspensorless embryos (EE), and suspensor-bearing embryos (SUS) were found to coexist in DH4079 microspore cultures (Supena et al., 2008). This was further refined in *B. napus* DH12075 microspore cultures, where besides EE and SUS, embryogenic compact and loose calli (CC and LC) were also described (Corral-Martinez et al., 2020). Embryo identity and fate were unambiguously assigned to these multicellular structures using embryo fluorescent reporters and time-lapse imaging (Li et al., 2014; Soriano et al., 2014; Corral-Martinez et al., 2020). However, not all these structures are able to develop into embryos. EE and SUS are highly embryogenic, producing most of the embryos, whereas LC and in particular CC are barely embryogenic. In this work we showed that very similar structures are also induced in DH4079 microspore cultures, demonstrating that the range of embryogenic types in DH4079 is wider than previously assumed. We observed EE, CC, and LC, anatomically equivalent to the structures found in DH12075. We also identified LBS, which resemble the early stages of DH12075 suspensor-bearing embryos (SUS), but growing slightly slower, having less cells and a less prominent suspensor at day 5, when observations were made. Since we did not track the fate of each structure, we erred on the side of caution and first considered LBS as a different type of structure. However, after completion of our study, we can reasonably assume that DH4079 and DH12075 embryogenic structures are equivalent based on their similar anatomies, degrees of exine rupture, levels of cell adhesion, general cell wall characteristics, and developmental fate.

Our study revealed equivalent differences between compact (EE and CC) and loose structures (LC and LBS/SUS), as well as similarities between the highly embryogenic (EE and LBS/SUS) and the barely embryogenic structures (CC and LC). These differences and similarities and their significance in the context of *in vitro* morphogenesis are discussed below.

### Different Microspore-Derived Structures Present Specific Cell Wall Profiles That Define Their Developmental Fate

**FIGURE 9:**
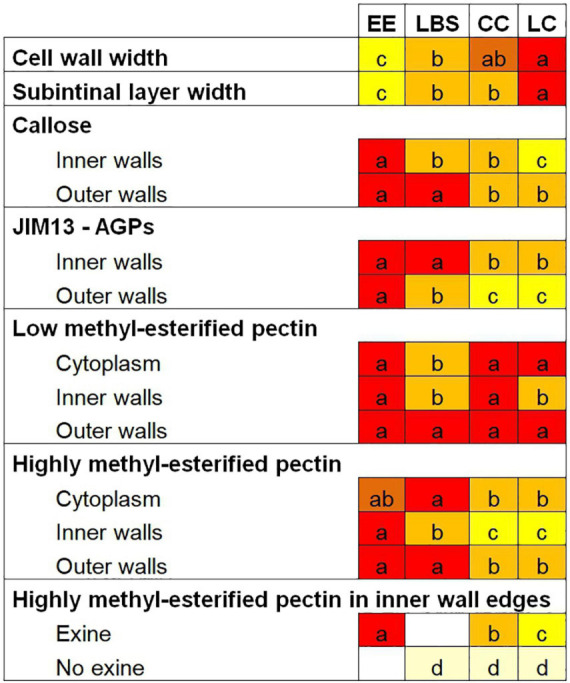
Summary of the cell wall composition characteristics of the different embryogenic microspore-derived structures. Different letters represent statistically significant differences in labeling density according to the LSD test, ranging from a (the highest labeling density) to d (the lowest labeling density). For a better comparison among structures, each letter is associated with a different background color.

Combining our data with that from previous studies (Li et al., 2014; Soriano et al., 2014; Corral-Martinez et al., 2020), we can conclude that the potential for differentiated embryo formation, even through different pathways (with or without a suspensor) correlates with the cell wall profile of the embryogenic structure. We examined these cell wall properties in detail by studying the thickness of outer cell walls (intine + subintinal layer), as well as the composition of inner and outer cell walls in terms of callose, cellulose, high and low methyl-esterified pectin, and AGPs. The most relevant differences are summarized in Figure 9. Our data indicate that EE structures, shown to be by far the most viable and embryo-producing structures in DH12075 (Corral-Martinez et al., 2020), had the thinnest walls of all the structures, but with the highest labeling density for almost all the antibodies used in this study. This suggests that this specific cell wall profile is the most favorable for cell proliferation and embryo progression. Conversely, CC, which produced almost no embryos and was the main source of calli (Corral-Martinez et al., 2020), had cell walls thicker than EE and an overall lower antibody signal, except for low methyl-esterified pectin, where a strong signal was observed in all cell walls. This cell wall profile, opposite to that of EE except for low methyl-esterified pectin, would confirm that embryo progression is favored by thin walls enriched in callose, AGPs, and high methyl-esterified pectin. It is interesting to note that the two compact structures, EE and CC, showed similarly high levels of low methyl-esterified pectin but completely different developmental fates. This assigns a role for low methyl-esterified pectin in the maintenance of cell adhesion and structural compactness, but not in the embryogenic competence.

Loose structures (LBS/SUS and LC), shown to have a very low viability in DH12075 (Corral-Martinez et al., 2020), showed the thickest cell walls and the lowest density of certain components in DH4079, especially in inner walls. It appears that these features accounted for the low levels of cell adhesion found between cells and eventually for their low viability. However, the few embryos they produced were all suspensor-bearing embryos (Corral-Martinez et al., 2020). It seems clear that this particular cell wall profile determined their developmental fate as barely viable structures, but with potential to become suspensor-bearing embryos. In particular, LC structures presented the widest cell walls and the lowest values for almost all the studied epitopes. They also have the lowest viability, with nearly 83% of them being arrested or undergoing only few cell divisions (Corral-Martinez et al., 2020). These cell wall properties, totally opposite to those of EE structures, could be defined as the less favorable for cell proliferation and embryo progression. In conclusion, we showed that each embryogenic structure has a specific cell wall profile that directly correlates with its ability to form embryos. The cell wall profile of each structure would define its developmental fate.

### The Newly Formed Cell Walls That Support Embryo Formation Are Thin, Plastic, and Callose-Rich

The inner cell walls formed in DH4079 embryogenic microspores between neighboring cells just upon induction are known to be plastic, irregular, incomplete, and rich in callose, an amorphous polymer that confers flexibility and fluidity to nascent cell plates (Samuels et al., 1995) and also to the cell walls of embryogenic microspores when it is not replaced by cellulose (Parra-Vega et al., 2015b). This combination of properties allows for cell expansion and growth (Parra-Vega et al., 2015b) and is found in the thin inner walls of highly embryogenic structures (EE and LBS/SUS) in both DH4079 (this work) and DH12075 (Corral-Martinez et al., 2020). In turn, these properties markedly differ from the thick walls found in barely embryogenic structures (CC and LC). Cell wall width is a crucial characteristic of multicellular structures. It affects not only the compactness of the structure, but also intercellular communication (Parra-Vega et al., 2015b), required to organize and functionally coordinate the role of cells within the same tissue or organ. Cell-to-cell communication proceeds through plasmodesmata, through which cells share chemical signals and form symplastic domains of shared communication. In zygotic embryogenesis, the first divided cells form a single symplast where cells are connected up to the heart-shaped embryo stage, when different symplasts are formed to start with organ differentiation (Kim and Zambryski, 2005). Intercellular connections maintain the identity of the early zygotic embryo, and it can be assumed that also of the microspore-derived embryo. During *in vitro* embryogenesis from somatic explants, embryogenic cells are isolated symplasmically from non-embryogenic cells through callose deposition in plasmodesmata (Godel-Jedrychowska et al., 2020). Intercellular connections are easier to establish in cells with thin and incomplete walls, where in addition to plasmodesmata, gaps, and fenestrae permit the contact of neighboring cytoplasms (Parra-Vega et al., 2015b). If inner walls become excessively thick, as in barely embryogenic structures (LC and CC), plasmodesmata would collapse and wall gaps would occlude, thereby reducing or preventing cell-to-cell communication. It is not surprising that those structures with thinner and more tightly connected cell walls (EE and LBE/SUS) have a higher probability to form a differentiated embryo (Corral-Martinez et al., 2020).

Here, we also examined the biological significance of the subintinal layer. This callose-rich layer is a good predictor for the ability to form differentiated embryos. It allows cells to create an impermeable layer underneath the intine and protects cells from osmotic changes in the *in vitro* culture environment, which has a positive impact on cell viability (Rivas-Sendra et al., 2019). In cell walls, callose tends to form highly hydrated but semipermeable gels, which are ideal physical and chemical barriers (Albersheim et al., 2011). During plant morphogenesis, callose is used by cells to isolate themselves from the surrounding environment, thereby preventing swelling or bursting and allowing them to express a new morphogenic program in isolation. Examples include natural processes such as the formation of male and female meiocytes (Heslop-Harrison and Mackenzie, 1967; Zhang et al., 2002; Abramova et al., 2003), *in vitro* organogenesis (Fortes et al., 2002), and somatic embryogenesis (Maheswaran and Williams, 1985; Dubois et al., 1991; Pedroso and Pais, 1992; You et al., 2006). Our observation that high levels of callose accumulated in the subintinal layer of outer cell walls of highly embryogenic structures (EE and LBS/SUS) is in line with these studies, and provides additional evidence in a different *in vitro* morphogenic system. Increased callose accumulation was observed even when the subintinal layer itself was not thicker than in barely embryogenic structures (LC and CC), which suggests that the level of callose accumulation rather than the thickness of the subintinal layer is the critical parameter of outer cell walls that supports microspore embryogenesis.

### High Levels of Highly Methyl-Esterified Pectin Are Necessary for Cell Wall Flexibility and Growth of Highly Embryogenic Structures

Microspores and pollen are covered by a protective layer, the exine, a key determinant for survival outside the plant. The exine is a relatively rigid coat whose flexibility is largely determined by the number and type of pollen apertures. However, during microspore embryogenesis this layer must eventually break to allow the release of the developing embryo. The specific place where exine breaks is critical for the fate of the released structure (Tang et al., 2013; Corral-Martinez et al., 2020). We showed that exine rupture occurs earlier and to a larger extent in LBS/SUS, and later in CC, LC, and EE. EE and LBS/SUS structures are by far the most embryogenic (Corral-Martinez et al., 2020), which indicates that the timing of exine rupture is not critical for embryo production. However, EE structures produced only suspensorless embryos, whereas LBS/SUS produced only suspensor-bearing embryos (Corral-Martinez et al., 2020), which points to a critical role for exine presence in the developmental pathway adopted by the structure.

Highly methyl-esterified pectin is more abundant in the intine of embryogenic microspores than in pollen-like structures (Corral-Martínez et al., 2019). Here, we found that highly methyl-esterified pectin was present in both inner walls and the intine, being more abundant in organized, highly embryogenic structures (EE and LBS/SUS; Figure 7). During somatic-type cytokinesis, pectin is synthesized in the Golgi stacks in a methyl-esterified form, and delivered through the cytoplasm *via* vesicles to the growing cell wall where it is de-esterified by pectin methyl-esterases (PMEs) and cross-linked by calcium, becoming stiff and thereby providing rigidity to the cell wall (Bou Daher and Braybrook, 2015). A similar mechanism has been described for pollen tube formation (Palin and Geitmann, 2012). During the first stages of microspore embryogenesis in *B. napus*, the expression levels of pectin methyl-esterase are low in cultures, and the levels of highly methyl-esterified pectin are high (Solis et al., 2016). In line with this, our results point to highly embryogenic structures (EE and LBS/SUS) as the main contributors to the high levels of highly methyl-esterified pectin described previously. The higher levels of highly methyl-esterified pectin, not yet de-esterified and cross-linked, would suggest that these structures are actively growing.

Surprisingly, we also found that highly methyl-esterified pectin is more abundant at the region of the edges of inner cell walls that connect with outer walls, including the adjacent cytoplasm, but much more abundant where these regions are covered by exine (Figure 8), suggesting a relationship between the presence of exine and the deposition of highly methyl-esterified pectin. The cytoplasm adjacent to these cell wall regions also had high levels of highly methyl-esterified pectin immunogold labeling that was proportional in all cases to the labeling found in the corresponding cell wall regions, suggesting that highly methyl-esterified pectin is actively synthesized at this stage and selectively transported to exine-covered regions. In pollen tubes, methyl-esterified pectin promotes the formation of loose walls at the tube tip that are necessary for expansion and tip growth (Li et al., 1994). Away from the tip, pectin de-esterification generates rigid walls that provide stability to the pollen tube. Consistent with this, the high levels of highly methyl-esterified pectin in the inner cell wall edge regions implies that these regions are growing more actively to adapt cell wall width to the increasing size of these growing cells, and that their pectin is not yet de-esterified. Thus, it is reasonable to propose that this pectin composition, typical of growing walls, is what permits the cell walls to produce the expanding force necessary for exine rupture. Indeed, it is common to find points of exine rupture precisely at these regions where inner walls connect with outer walls (arrowhead in Figure 1C). In turn, the regions devoid of exine with corresponding lower amounts of highly methyl-esterified pectin would reflect cell wall regions where expansion has already taken place and walls are transitioning to a more stable, non-growing state. This notion is supported by the observation that loose structures (LBS and LC) are the structures where less exine remains attached to the walls and lower levels of highly methyl-esterified pectin were observed.

### The Particular Combination of Pectin, Arabinogalactan Proteins, and Calcium Levels Determines the Developmental Fate of Exine-Enclosed and Loose Bicellular Structures/SUS Structures

The role of AGPs in microspore embryogenesis has been the object of study for many years. The AGP composition of the microspore walls changes drastically when microspores enter the embryogenic pathway, becoming rich in certain AGPs (Corral-Martínez et al., 2019). AGPs are known to play a role in different microspore culture systems, since their addition to the culture medium promotes embryogenesis, whereas their depletion inhibits it (Borderies et al., 2004; Tang et al., 2006; Corral-Martínez and Seguí-Simarro, 2014; Makowska et al., 2017). However, their specific role is still not known. Calcium accumulation is involved in the embryogenic commitment of microspores/pollen (Rivas-Sendra et al., 2017) and the relationship between calcium and AGPs during cell wall growth is well known. In growing pollen tubes, AGP depletion inhibits growth, whereas calcium influx promotes the delivery of exocytosis vesicles (Gaffal et al., 2007). Periplasmic AGPs can act as calcium capacitors, releasing calcium when the apoplast changes by the activation of H^+^-ATPs pumps due to the tensional force that occurs in the plasma membrane during cell growth (Lamport and Varnai, 2013; Lamport et al., 2018). Indeed, the ability of cell surface AGPs for binding and releasing apoplastic calcium was recently demonstrated (Lopez-Hernandez et al., 2020).

We showed that highly embryogenic structures (EE and LBS/SUS) have the highest density of JIM13-detected AGPs in both outer and inner cell walls and the highest levels of intracellular calcium soon after embryogenesis induction (Rivas-Sendra et al., 2017). According to the model of Lamport and Varnai (2013), AGPs would provide these structures with more capacity to release Ca^+2^ when needed for the microspore-to-embryo switch. When the plasma membrane stretches due to cell growth, calcium would be released by AGPs, thereby signaling the need for more cell wall synthesis and activating the delivery of exocytosis vesicles, as in pollen tube growth. This sets off a positive feedback loop that increases the amount of AGPs in the cell wall and in turn increases calcium. Calcium is also required for gelification of low methyl-esterified pectin to provide cellular adhesion (Palin and Geitmann, 2012; Bou Daher and Braybrook, 2015). In general, inner cell walls of compact structures (EE and CC) have a higher density of low methyl-esterified pectin, indicating their importance for cell adhesion. In addition, the combined high levels of AGPs, pectin, and calcium in EE structures would confer them unique properties to combine cell adhesion with high embryogenic potential.

Loose bicellular structures/SUS structures showed an unusual pattern of AGP accumulation. They have high amounts of AGPs in their inner walls and high amounts of both low and highly methyl-esterified pectin in their outer walls. The high degree of pectin esterification in outer walls and the detachment of the exine would provide flexibility to these walls, but the low degree of pectin esterification in inner cell walls would support cell adhesion. In other words, these structures are loose because the exine is not present to restrict their expansion and their outer walls allow for it, but the connection between cells is sufficient to maintain intercellular communication. This combination of cell wall properties provides the optimal conditions for their development as suspensor-bearing embryos, which would be nearly impossible with any other cell wall type. In support of this notion, LC structures, with similar pectin composition in inner and outer walls, were only able to produce suspensor-bearing embryos, although at a very low frequency (Corral-Martinez et al., 2020). In this case, the increased cell wall thickness and other differences in their cell wall components combined with their low viability would account for their low embryogenic response.

## Conclusion

Here we demonstrate that each type of structure has a different cell wall composition and architecture. The structures with the highest probability to become embryos (EE) have thin walls that allow them to keep cells close and communicated, a callose-rich subintinal layer that allows them to isolate from the environment and to create their own embryo identity, and a specific pectin and AGP composition that determines their development as embryos. Other structures with different cell wall profiles are also able to become embryos, but through different pathways and with lower rates of success (Corral-Martinez et al., 2020). There seems to be an optimal cell wall profile to become an embryo, but such profile is not the only possible. Other profiles would allow for embryogenesis, but less efficiently. From a developmental perspective, each structure seems to find its way to progress toward embryogenesis, but its success would be determined by the cell wall profile. The next question would be why each embryogenic structure has a different cell wall profile. We previously showed that the first cell walls formed by embryogenic microspores are morphologically altered and have an abnormal composition, rich in callose and deficient in cellulose, due to the partial impairment of conventional cytokinesis during the heat shock stage (Parra-Vega et al., 2015b). These problems are undergone by each microspore to different extents, leading to different cell walls with different properties in terms of adhesion, stiffness and permeability, among others. Each microspore may manage the situation differently, trying to compensate the deficiencies through different ways. This has been demonstrated to occur during pollen tube growth, a similar system where cell walls have untypically low cellulose contents. In this system, when cellulose deposition is compromised, alternative compensatory mechanisms for increased pectin synthesis and/or deposition are activated (Aouar et al., 2010; Palin and Geitmann, 2012). This way, pollen tubes are able to adapt their cell wall properties to a new scenario. Since both pollen tubes and cell walls of embryogenic microspores are formed from the microspore/pollen plasma membrane and cell wall, their initial composition and chemical environment should be similar. Given the similarities between both cell walls, it is plausible to propose that similar, pectin-based compensatory mechanisms may operate in both cases. In conclusion, this study provides relevant data to further understand the role of cell wall during the initial stages of microspore embryogenesis, and how different cell wall profiles are related to different developmental fates.

## Data Availability Statement

The original contributions presented in the study are included in the article/supplementary material, further inquiries can be directed to the corresponding author.

## Author Contributions

PC-M, KB, and JS-S: conceptualization and methodology. PC-M, CC-F, and RM: data curation, formal analysis, and investigation. PC-M, CC-F, and JS-S: visualization. PC-M and JS-S: supervision. CC-F and JS-S: writing – original draft preparation. JS-S and KB: writing – review and editing. All authors contributed to the article and approved the submitted version.

## Conflict of Interest

The authors declare that the research was conducted in the absence of any commercial or financial relationships that could be construed as a potential conflict of interest.

## Publisher’s Note

All claims expressed in this article are solely those of the authors and do not necessarily represent those of their affiliated organizations, or those of the publisher, the editors and the reviewers. Any product that may be evaluated in this article, or claim that may be made by its manufacturer, is not guaranteed or endorsed by the publisher.
